# It’s a thin line: development and validation of Dixon MRI-based semi-quantitative assessment of stress-related bone marrow edema in the wrists of young gymnasts and non-gymnasts

**DOI:** 10.1007/s00330-019-06446-8

**Published:** 2019-11-27

**Authors:** L. S. Kox, R. B. J. Kraan, V. Mazzoli, M. A. Mens, G. M. J. J. Kerkhoffs, A. J. Nederveen, M. Maas

**Affiliations:** 1 Department of Radiology and Nuclear Medicine, University of Amsterdam, Amsterdam Movement Sciences, Amsterdam UMC, location AMC, G1-229, Meibergdreef 9, 1105 AZ Amsterdam, The Netherlands; 2 Academic Center for Evidence-based Sports medicine (ACES), Amsterdam, The Netherlands; 3 Amsterdam Collaboration for Health and Safety in Sports (ACHSS), International Olympic Committee (IOC), Research Center AMC/VUmc, Amsterdam, The Netherlands; 4 Department of Radiology, Stanford University, Stanford, CA USA; 5 Department of Orthopedic Surgery, Amsterdam UMC, location AMC, Amsterdam, The Netherlands

**Keywords:** Wrist injuries, Growth plate, Athletic injuries, Gymnastics, Magnetic resonance imaging

## Abstract

**Purpose:**

To assess reliability and clinical utility of evaluating stress-related metaphyseal water distribution using a semi-quantitative Dixon MRI-based method for early diagnosis of physeal stress injuries in adolescent gymnasts.

**Methods:**

Twenty-four gymnasts with clinically suspected overuse injury of the distal radial physis, 18 asymptomatic gymnasts, and 24 non-gymnast controls aged 12 ± 1.5 years prospectively underwent hand radiographs and 3T MRI of the wrist including coronal T1-weighted and T2-weighted Dixon sequences. Two raters measured metaphyseal water signal fraction in 13 radial and ulnar regions of interest (ROI). Inter- and intrarater reliability, interslice (between 3 middle radial slices), and inter-ROI (between 3 ROIs on same level) reliability were assessed using intraclass correlation coefficients (ICC). Water signal fractions and their within-person ratios in distal versus most proximal ROIs were compared between groups using one-way analysis of variance.

**Results:**

Inter- and intrarater ICCs were 0.79–0.99 and 0.94–1.0 for T1-weighted, and 0.88–1.0 and 0.88–1.0 for T2-weighted Dixon. Interslice and inter-ROI ICCs were 0.55–0.94 and 0.95–0.97 for T1-weighted, and 0.70–0.96 and 0.96–0.97 for T2-weighted Dixon. Metaphyseal water signal fraction in symptomatic gymnasts was higher in six distal ROIs compared with asymptomatic gymnasts and in nine ROIs compared with non-gymnasts (*p* < 0.05). Metaphyseal water score (ratio of distal versus most proximal ROIs) was 1.61 in symptomatic gymnasts and 1.35 in asymptomatic gymnasts on T2-weighted Dixon (*p* < 0.05).

**Conclusion:**

Semi-quantitative Dixon MRI-based water signal fraction assessment has good to excellent reproducibility and shows increased metaphyseal water scores in symptomatic gymnasts compared with asymptomatic gymnastic peers.

**Key Points:**

• *The proposed Dixon MRI-based semi-quantitative method for assessment of metaphyseal bone marrow water content is reliable, with off-the-shelf availability and short scan times.*

• *The metaphyseal water score allows comparisons between gymnasts using a within-person reference area for unaffected metaphyseal bone.*

• *As metaphyseal water score was increased in symptomatic gymnasts compared with asymptomatic gymnasts, this semi-quantitative method can potentially be used as an indicator of bone marrow edema in the early diagnosis of gymnastic physeal stress injury.*

**Electronic supplementary material:**

The online version of this article (10.1007/s00330-019-06446-8) contains supplementary material, which is available to authorized users.

## Introduction

During the growth spurt, young athletes are vulnerable to overuse injuries and fractures [1–3]. Growth plates are potential sites for stress injury, like the distal radial physis in young gymnasts [4, 5]. This overuse injury is characterized by wrist pain, which up to 79% of young gymnasts report [6]. Radial physeal stress injury has been linked to inhibited radial growth and positive ulnar variance, sometimes with long-term consequences like ulnar impaction syndrome and triangular fibrocartilage complex degeneration [7–10]. Early diagnosis of stress injuries is essential to minimize risk of degenerative conditions and recovery time.

Magnetic resonance imaging (MRI) offers detailed imaging of early-stage injury by displaying metaphyseal bone bruising [11, 12]. However, in children, bone marrow edema can be difficult to differentiate from physiological, maturation-induced high signal areas on T2-weighted MRI [13, 14]. Such edematous changes in hands and other areas can occur in asymptomatic young athletes [15–18] and asymptomatic active children [19, 20]. These changes are hypothesized to arise from bone contusion [17] and bone remodeling following stress-induced hyperperfusion [15], possibly in response to growth-related biomechanical stress [13].

For qualitative MRI assessment of wrist physes, a validated protocol is available [21]. Additional quantification of metaphyseal water content may be able to detect early edematous changes of the bone marrow that are not (yet) identifiable during qualitative MRI assessment and may therefore improve assessment of maturation-related versus stress-induced bone marrow edema in symptomatic gymnasts. Furthermore, we assume that water content is further increased in gymnasts with physeal stress injury compared with asymptomatic gymnasts, and therefore that water content assessment can be valuable to confirm or exclude the presence of early stress-related injury.

Dixon chemical-shift imaging can be used for fat suppression in extremities [22], but also for fat quantification in bone [23]. Similarly, water signal fraction can be calculated from the acquired images. The primary spongiosa of newly formed metaphyseal bone adjacent to the physis appears on T2-weighted scans as a high-signal area [24], and water signal fraction in this epimetaphyseal region thus likely is high during both normal maturation and (early-stage) physeal stress injury. However, we hypothesize that this metaphyseal water signal fraction is higher in gymnasts compared with non-gymnasts, that this difference is decreasing towards the diaphysis, and that the metadiaphyseal bone even further proximal can serve as a reference area, likely to remain unaffected by physeal stress injury.

This study’s purpose was to explore the magnitude and distribution of metaphyseal water content in the distal radius and ulna of young gymnasts with wrist pain, asymptomatic gymnasts and non-gymnasts, and to assess the reliability and clinical utility of a semi-quantitative MRI method for water signal fraction measurement. We hypothesized that the proposed method is reliable and useful for detecting early differences in metaphyseal water content that constitute the thin line between these three groups, and that water signal fractions are highest in symptomatic gymnasts and lowest in controls.

## Materials and methods

### Study design

This prospective observational study was performed according to the Declaration of Helsinki and approved by our internal review board (reference no. 2014_382). Participants between 12 and 18 years old were included from June 2015 until November 2017. Symptomatic gymnasts defined as gymnasts with clinically suspected growth plate stress injury of the wrist were referred by (sports) physicians. Asymptomatic gymnasts and non-gymnast controls were recruited through gymnastics clubs, the bring-a-friend strategy, and via notices within our institution. Written informed consent was obtained from all participants and their parents or guardians prior to participation.

### Study population

We included 66 participants (mean age, 14.2 years; range, 12.0–17.8 years). These comprised 24 symptomatic gymnasts (mean age, 14.4 years; range, 12.1–16.9 years), as well as 18 asymptomatic gymnasts (mean age, 14.4 years; range, 12.1–17.8 years) and 24 non-gymnasts (mean age, 13.7 years; range, 12.0–17.0 years). Exclusion criteria were history of past fracture, wrist surgery or infection, growth disorder, systemic or oncological disease involving the musculoskeletal system, and fully closed growth plate on hand radiograph. Participants filled out a questionnaire on wrist pain, demographic, and sports characteristics. Gymnasts had performed their sport for at least 1 year and up to 6 months or less prior to study participation.

### Imaging

Posterior-anterior radiographs with a 1.30-m focus detector distance of one (symptomatic) hand and wrist were obtained. MRI of the (symptomatic) wrist was performed in a feet-first, supine position with arms resting alongside the body on a 3.0T MRI scanner (Ingenia, Philips Healthcare) using a dedicated eight-channel receive-only wrist coil. MRI included the qualitative protocol for regular patient care (approximately 20 min) and additional quantitative coronal turbo spin-echo (TSE) T1-weighted and TSE T2-weighted 2-point Dixon series (Table 1).

**Table 1:** MRI parameters

Sequence	T1-weighted Dixon	T2-weighted Dixon
Plane	Coronal	Coronal
Repetition time (ms)	639	2500
Echo time (ms)	(1) 20 (2) 21	(1) 70 (2) 71
Flip angle (degrees)	90	90
Slice thickness (mm)	2	2
Field of view (mm)	100 × 100	100 × 100
Matrix	312 × 216	312 × 235
Spatial resolution (mm)	0.32 × 0.46 × 2	0.32 × 0.43 × 2
Water-fat shift (pixels)	1.6	1.5
Scan time (minutes)	04:47	04:10

### Post-processing

Bone age was determined on the radiographs using validated software (BoneXpert, v2.0.1.3; Visiana, www.BoneXpert.com) [25]. Dixon MRI scans were reconstructed into fat-only and water-only images using the vendor’s standard software, and subsequently blinded and transformed into series of calculated images with every voxel representing the water signal fraction $\left(\frac{water- only}{\left( water- only+ fat- only\right)}\right)$, using MATLAB (MATLAB and Image Toolbox Release 2017b, the MathWorks, Inc.).

### Water signal fraction measurement

**Fig. 1:**
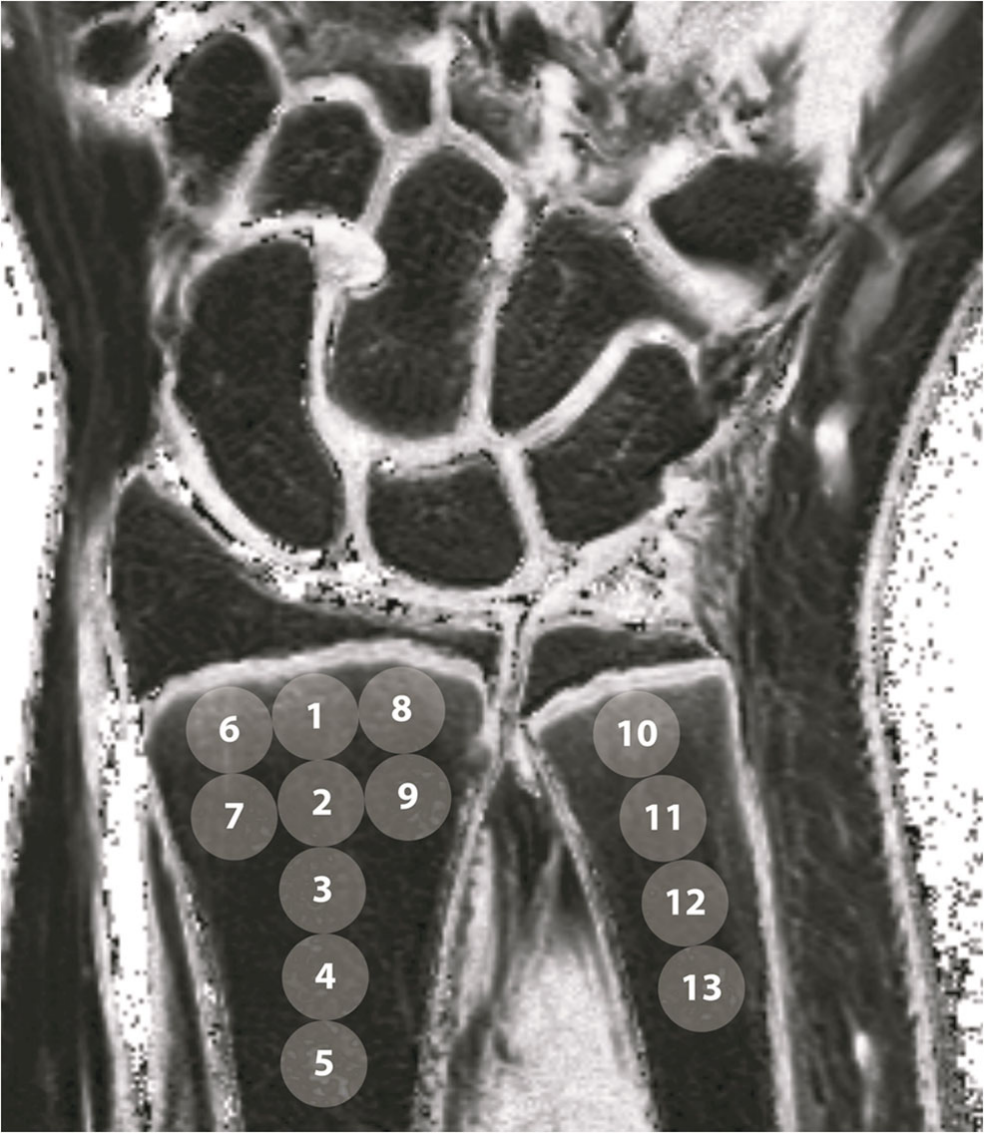
Placement of regions of interest on post-processed coronal T1-weighted and T2-weighted Dixon images. ROIs 1, 6, 8, and 10 were placed directly adjacent to the physis, representing most distal 0–5 mm of the metaphysis

The primary outcome measure was distribution and magnitude of metaphyseal water signal fraction in the radius and ulna. Two musculoskeletal radiology research physicians independently measured metaphyseal water signal fraction on three slices showing the radial maximal width and on the slice showing the ulnar maximal width. Thirteen regions of interest (ROIs) with a 5-mm diameter were drawn using ImageJ (v1.50i, US National Institutes of Health, https://imagej.nih.gov/ij/) (Fig. 1). ROIs ranged from the epimetaphysis 0–5 mm proximal to the physis, to the metadiaphysis 20–25 mm proximal to the physis in order to evaluate metaphyseal water signal fraction distribution. After 20 training measurements, both raters performed measurements in 25 participants independently on T1-weighted and T2-weighted images and interrater agreement was determined. In case of good interrater reliability (intraclass correlation coefficient ≥ 0.75), one rater performed measurements in all participants for further analysis.

### Statistical analysis

Intra- and interrater reliability was determined by calculating the intraclass correlation coefficient (ICC) for absolute agreement for each ROI, per sequence, using a two-way random analysis of variance (ANOVA) model (case 2, ICC(2,1)) and the coefficient of variation (CV), defined as the standard deviation of the paired differences divided by the population mean for each ROI. Difference of mean paired differences from zero was assessed using the one-paired *t* test. Levels of agreement were defined as ICC < 0.5 = poor, ICC 0.5–0.75 = moderate, ICC 0.75–0.9 = good, ICC > 0.9 = excellent [26].

To determine inter-slice reliability, the ICC for absolute agreement between water signal fraction measured on the three middle slices of the radius was calculated using a two-way mixed ANOVA model. Inter-ROI reliability was determined by calculating the ICC for absolute agreement between the mean of ROIs 1, 6, and 8 and ROI 1, and between the mean of ROIs 2, 7, and 9 and ROI 2, using a two-way mixed ANOVA model. Correlation of water signal fraction values between sequences was determined using linear regression.

Ratios were calculated for water signal fraction of the distal, epimetaphyseal ROIs (ROIs 1 and 2 for the radius, ROI 10 for the ulna) compared with the most proximal, diametaphyseal ROI in the same bone (ROI 5 for the radius, ROI 13 for the ulna), which was considered the “reference ROI.” These ratios will further be referred to as “metaphyseal water score.” Between-group differences in participant characteristics and water signal fractions were evaluated using one-way ANOVA, or the Kruskal Wallis test for not normally divided data. In case of significant differences, Tukey or Games-Howell post hoc testing (for normally divided data) or Dunn-Bonferroni post hoc testing (for not normally divided data) was performed to identify differing groups.

Data analysis was performed using IBM SPSS Statistics (v24.0, 2016). A sample size calculation based on an expected effect size for water signal fraction ratio of 0.20 leads to a preferred sample size of 18 participants per group for a power of 80%. *p* < 0.05 was considered statistically significant.

## Results

### Participant characteristics

Participant demographics are reported in Table 2. Although skeletal and calendar ages did not differ between groups, the ratio of skeletal age compared with calendar age differed significantly between girls in both gymnast groups compared with the non-gymnast control group (Table 2).

**Table 2:** Participant characteristics

Sex	Symptomatic gymnasts		Asymptomatic gymnasts		Non-gymnast controls	
	Female (*n* = 12)	Male (*n* = 12)	Female (*n* = 9)	Male (*n* = 9)	Female (*n* = 12)	Male (*n* = 12)
Calendar age (years)	14.3 ± 1.6	14.5 ± 1.1	14.5 ± 1.5	14.4 ± 1.9	13.8 ± 1.2	13.6 ± 1.6
Skeletal age (years)	12.6 ± 1.0	13.9 ± 1.5	12.1 ± 1.4	13.7 ± 2.4	13.7 ± 1.8	13.5 ± 1.9
$\frac{Skeletal\ age}{calendar\ age}$	0.88 ± 0.10*	0.96 ± 0.05	0.84 ± 0.06^§^	0.95 ± 0.06	0.99 ± 0.09	1.0 ± 0.07
Height (cm)	155 ± 11	162 ± 11	158 ± 9	159 ± 9	164 ± 7	164 ± 15
Weight (kg)	43.8 ± 9.5	50.5 ± 9.0	45.7 ± 8.3	48.8 ± 8.9	51.8 ± 7.9	51.4 ± 15.6
Age at start of gymnastics training (years)	5.9 ± 1.2	5.4 ± 1.4	5.4 ± 1.3	6.4 ± 1.3	NA	NA
Gymnastics training hours/week	19 (12–29)	23 (14–27)	31 (13–34)	14 (10–21)	NA	NA
Gymnastics experience (years)	8.5 ± 2.2	9.1 ± 1.7	9.0 ± 1.0	8.0 ± 2.6	NA	NA
Gymnastics training level	Elite (*n* = 11) Non-elite (*n* = 1)	Elite (*n* = 12) Non-elite (*n* = 0)	Elite (*n* = 9) Non-elite (*n* = 0)	Elite (*n* = 7) Non-elite (*n* = 2)	NA	NA

Data are presented as mean ± standard deviation or as median with (interquartile range)

*NA*, not applicable

*Difference of symptomatic gymnasts compared with non-gymnast controls significant at level *p* < 0.05

^§^Difference of asymptomatic gymnasts compared with non-gymnast controls significant at level *p* < 0.05

### Inter- and intrarater reliability

For water signal fraction measurements on T1-weighted Dixon, interrater agreement was good to excellent (ICC, 0.79–0.99; CV, 2.6–27.7%) and intrarater agreement was excellent (ICC, 0.91–1.0; CV, 2.1–10.9%) for individual ROIs in the radius and ulna (Supplementary Table 1). Interrater agreement was good to excellent (ICC, 0.88–1.0; CV, 1.9–19.7%) and intrarater agreement was good to excellent (ICC, 0.88–1.0; CV, 3.8–18.7%) on T2-weighted Dixon as well (Supplementary Table 2). Since these outcomes indicated good to excellent interrater reliability for the majority of ROIs, measurements of one observer were used for further analysis.

### Inter-slice and inter-ROI reliability

The ICC for inter-slice agreement between ROIs on the three middle slices of the radius was moderate to excellent on T1-weighted Dixon (ICC, 0.55–0.94) and T2-weighted Dixon (ICC, 0.70–0.96) (Supplementary Table 3).

Inter-ROI agreement for ROI 1 and the mean of ROIs 1, 6, and 8 was excellent on T1-weighted and T2-weighted Dixon (respective ICCs, 0.97 and 0.96). Inter-ROI agreement between ROI 2 and the mean of ROIs 2, 7, and 9 was also excellent on T1-weighted and T2-weighted Dixon (ICCs, 0.95 and 0.97, respectively) (Supplementary Table 4). As the agreement between these ROIs was excellent, we decided to focus on ROIs 1–5 for the radius.

### Correlation between sequences

For all ROIs, measurements on T1-weighted and T2-weighted Dixon showed a linear correlation with slopes significantly different from one, with consistently higher water signal fractions on T2-weighted Dixon compared with T1-weighted Dixon (Supplementary Fig. 1). ROIs on T1-weighted images demonstrated smaller standard deviations and therefore a slightly higher effect size.

### Metaphyseal water signal fraction

**Fig. 2:**
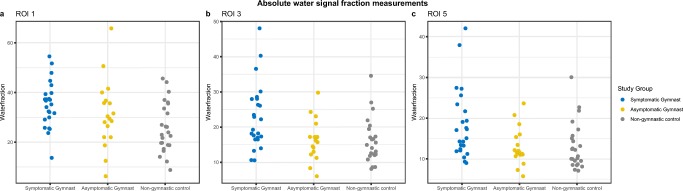
Scatterplots of absolute water signal fraction measured on T2-weighted Dixon images of ROI 1 (**a**), ROI 3 (**b**), and ROI 5 (**c**) in the three study groups

Water signal fraction was significantly higher in symptomatic gymnasts compared with asymptomatic gymnasts in 3 distal radial ROIs (ROIs 2–4) on T1-weighted or T2-weighted Dixon images, but not in the metadiaphyseal reference area (ROI5) (Table 3). In all radial ROIs on T1-weighted Dixon images, and in the ulnar epimetaphyseal ROI 10, water signal fraction was significantly higher in symptomatic gymnasts compared with non-gymnasts on both sequences. No significant differences were observed in any of the ROIs between asymptomatic and non-gymnasts. Scatterplots of absolute water signal fraction measurements in ROIs 1, 3, and 5 on T2-weiged Dixon images in the three study groups can be found in Fig. 2.

**Table 3:** Water signal fraction measurements per group and per sequence in radius and ulna

	Symptomatic gymnasts		Asymptomatic gymnasts		Non-gymnast controls	
	T1w Dixon	T2w Dixon	T1w Dixon	T2w Dixon	T1w Dixon	T2w Dixon
Radius						
ROI 1	24.8 ± 6.2*	35.2 ± 9.3*	24.0 ± 11.9	31.3 ± 13.7	18.2 ± 6.5	25.8 ± 10.1
ROI 2	15.8 ± 5.1*^†^	22.9 ± 9.3*^†^	12.1 ± 3.0	16.4 ± 5.7	11.7 ± 3.2	15.9 ± 6.4
ROI 3	13.1 ± 4.3*^†^	18.4 ± 8.6*^†^	10.4 ± 2.3	12.9 ± 4.5	10.2 ± 2.8	13.1 ± 5.7
ROI 4	11.7 ± 2.9*^†^	15.5 ± 5.4*	9.7 ± 1.9	12.2 ± 3.7	9.6 ± 2.3	11.7 ± 4.4
ROI 5	11.0 ± 2.6*	14.1 ± 4.4*	9.8 ± 1.9	12.1 ± 3.7	9.1 ± 1.9	10.9 ± 3.6
Ulna						
ROI 10	26.4 ± 4.7*	37.0 ± 7.5*	24.0 ± 7.3	31.8 ± 10.1	20.9 ± 7.2	28.8 ± 10.9
ROI 11	15.8 ± 3.5	22.2 ± 6.0	14.0 ± 3.5	18.3 ± 5.8	13.6 ± 3.9	18.3 ± 7.3
ROI 12	12.6 ± 2.9	16.7 ± 4.7	11.2 ± 2.8	14.0 ± 5.2	11.3 ± 2.9	14.1 ± 5.1
ROI 13**^§^	11.0 ± 2.5	14.2 ± 4.4	10.1 ± 2.8	12.4 ± 4.7	10.0 ± 2.4	11.8 ± 4.1

Data are presented as mean ± standard deviation

*T1w*, T1-weighted; *T2w*, T2-weighted; *ROI*, region of interest

*Difference of symptomatic gymnasts compared with non-gymnast controls significant at level *p* < 0.05

^†^Difference of symptomatic gymnasts compared with asymptomatic gymnasts significant at level *p* < 0.05

**One case was excluded for analysis of water signal fraction measured on T1-weighted Dixon because of partial overlap with metaphyseal cortex

^§^Two cases were excluded for analysis of water signal fraction measured on T2-weighted Dixon because of partial overlap with metaphyseal cortex

**Fig. 3:**
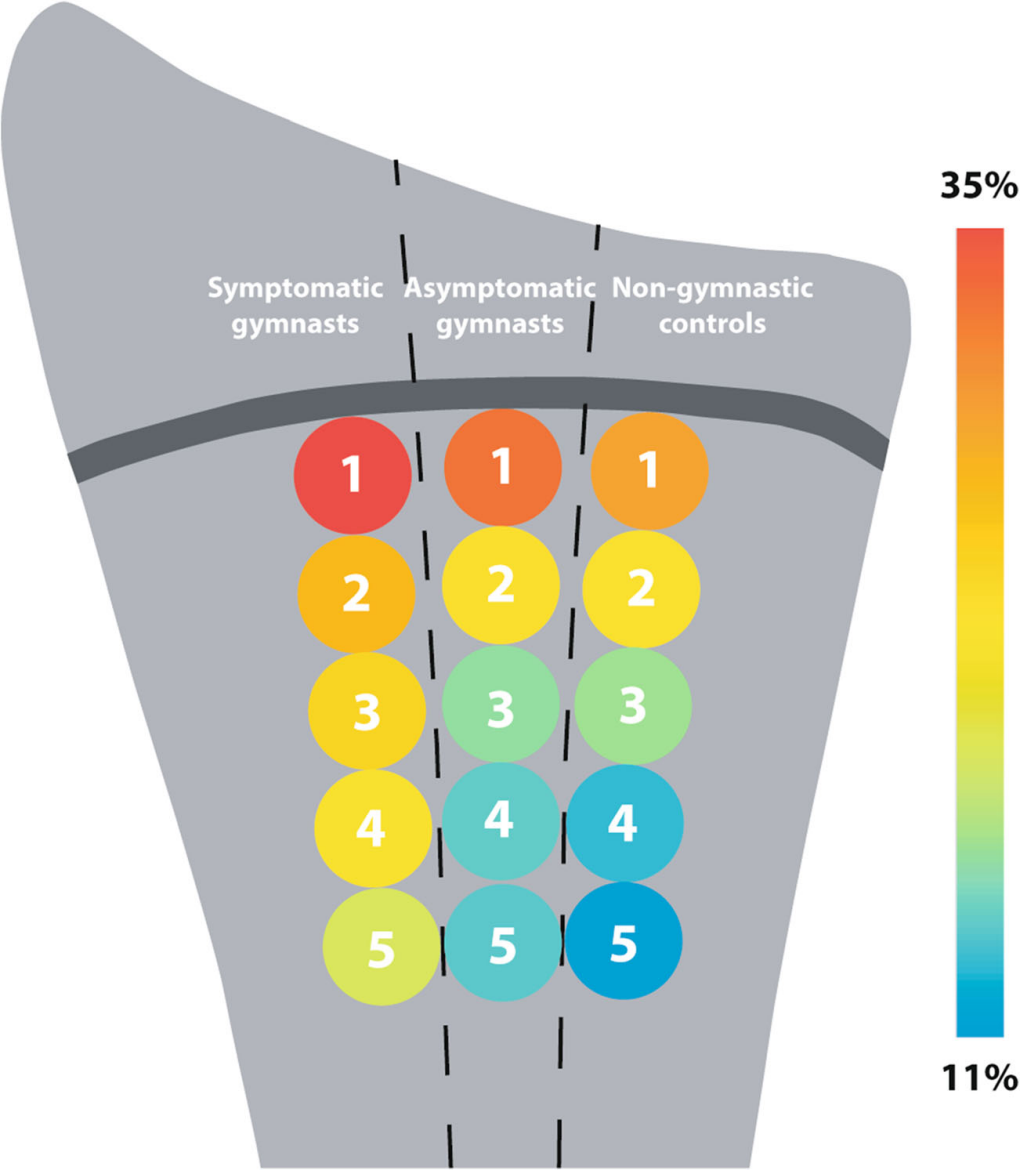
Schematic overview of the mean relative water signal fractions in five radial regions of interest for each participant group. Regions of interest are indicated by their number (1–5) and colors represent the magnitude of the mean water signal fraction for each region of interest

In the radius, the metaphyseal water score (the ratio of the water signal fraction of epimetaphyseal ROI 2 versus the water signal fraction of metadiaphyseal ROI 5) was significantly higher in symptomatic gymnasts compared with asymptomatic gymnasts on both sequences (Table 4, Fig. 3). On T1-weighted Dixon images, this ratio was also higher in symptomatic gymnasts compared with non-gymnasts. In the ulna, the metaphyseal water score of the epimetaphyseal ROI 10 versus the metadiaphyseal ROI 13 was significantly higher in symptomatic gymnasts compared with non-gymnasts on T1-weighted Dixon images (Table 4). No significant differences were observed in ratios between asymptomatic and non-gymnastics on T1- and T2-weighted Dixon images in both the radius and the ulna.

**Table 4:** Ratios of water signal fractions of ROI 1 and ROI 2 versus ROI 5 on the middle slice of T1-weighted and T2-weighted Dixon images

	Sequence	Ratio			*p* value
		Symptomatic gymnasts (*n* = 24)	Asymptomatic gymnasts (*n* = 18)	Non-gymnast controls (*n* = 24)	
Radius					
ROI 1 vs. ROI 5	T1w Dixon	2.3 ± 0.4	2.4 ± 1.2	2.0 ± 0.4	0.09
	T2w Dixon	2.6 ± 0.6	2.5 ± 0.8	2.4 ± 0.6	0.52
ROI 2 vs. ROI 5	T1w Dixon	1.4 ± 0.3*^†^	1.2 ± 0.2	1.3 ± 0.2	0.005
	T2w Dixon	1.6 ± 0.3^†^	1.4 ± 0.2	1.5 ± 0.2	0.01
Ulna					
ROI 10 vs. ROI 13	T1w Dixon**	2.5 ± 0.5*	2.4 ± 0.7	2.1 ± 0.4	0.02
	T2w Dixon^††^	2.8 ± 0.6	2.7 ± 0.9	2.5 ± 0.6	0.36
ROI 11 vs. ROI 13	T1w Dixon**	1.5 ± 0.2	1.4 ± 0.3	1.4 ± 0.2	0.29
	T2w Dixon^††^	1.6 ± 0.3	1.6 ± 0.6	1.6 ± 0.3	0.97

Data are presented as mean ± standard deviation

*ROI*, region of interest; *T1w*, T1-weighted; *T2w*, T2-weighted

*Difference of symptomatic gymnasts compared with non-gymnast controls significant in one-way ANOVA

^†^Difference of symptomatic gymnasts compared with asymptomatic gymnasts significant in one-way ANOVA

**One case was excluded for analysis of water signal fraction measured on T1 Dixon because ROI 13 did not fully fit in the field of view

^††^Two cases were excluded for analysis of water signal fraction measured on T2 Dixon because ROI 13 did not fully fit in the field of view

## Discussion

### Main findings

We found increased radial and ulnar metaphyseal water signal fractions in symptomatic gymnasts compared with asymptomatic gymnasts and non-gymnasts, using a reliable semi-quantitative Dixon MRI-based method. The ratio of radial metaphyseal water signal fraction in areas 5–10 mm versus 20–25 mm proximal to the physis was higher in symptomatic gymnasts compared with asymptomatic gymnasts, while their gymnastics level, training hours, and water signal fraction 20–25 mm proximal to the physis did not differ significantly.

### Proposed semi-quantitative method

Inter- and intrarater agreement was comparable for T1-weighted and T2-weighted Dixon sequences. The consistent discrepancy of water signal fraction on T1- and T2-weighted images most likely originates from the sequences’ difference in T1- and T2-weighting. Although the effect size of the T1-weighted sequence was slightly larger, significant differences were present in both T1- and T2-weighted images, and as T2-weighted Dixon allows both morphological and semi-quantitative image evaluation, we recommend its use for this measurement method to minimize scan time.

Even with good inter-ROI and interslice reliability, water signal fraction measurement in single ROIs may be difficult to reproduce and compare in clinical practice, as reference values have not yet been established and will differ among MRI scanners and protocols. We therefore evaluated the clinical utility of a “metaphyseal water score”: the ratio of the epimetaphyseal area 5–10 mm proximal to the radial physis (ROI2) versus a within-person reference area 20–25 mm proximal to the physis (ROI5). This ratio also showed between-group differences, and is presumably less sensitive to scanner-dependent bias than single-ROI-based water signal fractions when reproduced at other institutions using off-the-shelf Dixon MRI sequences. However, effect size of the metaphyseal water score was smaller compared with absolute water signal fractions, and therefore, its applicability for evaluation of injury severity, prognosis, and relationship with gymnastics training intensity needs further evaluation in larger athlete and non-athlete populations.

### Potential mechanisms

The overall increased metaphyseal water signal fractions in symptomatic gymnasts compared with both other groups suggest that higher water signal fraction is indicative of physeal stress injury. However, intensive sports performance, stress injury, and maturation likely all contribute to edema-like changes, and therefore, the line is thin between injury-related edema and physiological increase in metaphyseal water content.

Residual red bone marrow in asymptomatic active children can cause signal intensity changes [19, 20], like high signal intensity on T2-weighted MRI, easily mistaken for abnormalities [27]. Heterogeneous red bone marrow is a common MRI finding when small areas have not yet undergone the physiologic conversion to yellow bone marrow that starts distally in the bone during late childhood [28]. The marrow’s subsequent increase in fat content and decrease in water content [24] likely affect water signal fraction [28]. In this study, participants were therefore matched on skeletal age prior to inclusion to minimize potential interference of maturation with the study’s results.

Additionally, focal periphyseal edema can be seen adjacent to physes in response to growth-induced biomechanical stress in the area of initial physeal closure [29]. The ROIs in this epimetaphyseal region showed highest water signal fractions in all groups, and largest inter- and intrarater variability, suggesting more proximal areas like ROI 2 to be more suitable for identifying stress injury.

Young gymnasts often show attenuated growth and delayed menarche compared with non-gymnasts [30, 31], and in our study, despite the absence of significant differences in absolute skeletal or calendar age between the study groups, skeletal in relation to calendar age was significantly younger in female gymnasts compared with non-gymnasts. Although the exact relationship between maturation status and changes in bone marrow composition is unclear, we postulate that delayed maturation in—especially female—gymnasts may cause a stress-induced marrow shift delay, with relatively higher percentages of red marrow contributing to increased metaphyseal water signal fractions compared with non-gymnasts. In line with this, we found that water signal fraction in the radial reference area (ROI 5) was significantly higher in symptomatic gymnasts compared with non-gymnasts, but not to asymptomatic gymnasts. However, contradictory to our expectations, the increases in water signal fraction of the radial reference ROI in asymptomatic gymnasts compared with non-gymnasts were not significantly different. Future studies with larger sample sizes should explore if the absence of this difference between asymptomatic and non-gymnasts is to be attributed to the relative small study population in this explorative study.

Finally, distal radial bone mineral content and bone mineral density can increase after wrist-loading sports performance during youth [32]. Bone composition changes may influence the bone’s water and fat distributions and MRI signal derived from these components. Effects on ulnar mineralization status have not been documented, but as physeal stress injury reportedly occurs mainly in the radius because of its major weight-bearing function in the pediatric wrist [7], these load-induced changes may be more distinct in the radius.

As the metadiaphyseal ROI 5 showed higher water signal fractions in symptomatic gymnasts compared with non-gymnasts—but not asymptomatic gymnasts—increased metaphyseal water content more proximal to the physis may (partly) result from gymnastics practice. However, asymptomatic gymnasts showed no increased water signal fractions compared with non-gymnasts in any ROIs in the radius and ulna. Symptomatic and asymptomatic gymnasts had a similar training intensity, and therefore, we assume that the significant increase in water signal fractions in symptomatic gymnasts compared with non-gymnasts in nearly all radial ROIs, and one epimetaphyseal ulnar ROI, cannot be merely attributed to (physiological) changes in bone mineral content and density.

Considering these findings and potential gymnastics-induced bone composition changes, we recommend comparing metaphyseal edema scores of symptomatic gymnasts with those of asymptomatic gymnasts instead of non-gymnasts. This comparison is also relevant for clinical purposes; as in daily practice, the method is aimed to confirm or exclude the presence of early stress-related changes in gymnasts. The absence of between-group differences in skeletal age and training intensity suggests that the observed differences in absolute water signal fraction in several ROIs and metaphyseal water score between symptomatic and asymptomatic gymnasts are the result of physeal stress injury. This indicates that the proposed method can aid in detecting early stress-related edematous changes that indicate the presence of physeal stress injury. Further studies should explore the method’s feasibility for assessment of injury severity and for using it for following up stress injuries.

## Strengths and limitations

We included gymnasts and non-gymnasts to evaluate both stress-induced and maturation-induced changes. Observers performed training measurements to avoid learning curve effects. The metaphyseal water score’s areas (ROIs 2 and 5) demonstrated excellent inter- and intrarater reliability with limited variability.

However, the group differences presented here warrant future studies to determine cutoff values for individual gymnasts. Additionally, although Dixon sequences with two echo-times are considered adequate for water/fat separation, usually more echo-times are used for quantification purposes. For this pediatric population, however, the accompanying increase in scan time required for such sequences was considered undesirable. Nevertheless, this study provides a reliable semi-quantitative method with minimal patient and physician burden.

## Conclusion

Dixon MRI-based semi-quantitative assessment of metaphyseal water content can reliably show differences in water signal fraction between symptomatic gymnasts, asymptomatic gymnasts, and non-gymnasts. Symptomatic gymnasts showed increased radial metaphyseal water scores compared with asymptomatic gymnasts, illustrating that the proposed method can be useful in the assessment of stress-related bone marrow edema, despite the thin line between injury and physiological stress-related bone marrow edema.

## Electronic supplementary material

ESM 1 (DOCX 205 kb)

## Abbreviations

CV: Coefficient of variation
ICC: Intraclass correlation coefficient
TSE: Turbo spin-echo
