# Supplementary material for: It’s a thin line: development and validation of Dixon MRI-based semi-quantitative assessment of stress-related bone marrow edema in the wrists of young gymnasts and non-gymnasts
Source: Eur Radiol. 2019 Nov 27;30(3):1534–43. doi: 10.1007/s00330-019-06446-8 (PMC7033069; doi:10.1007/s00330-019-06446-8)
Supplement: Supplementary file 1 — (DOCX 205 kb) [file 330_2019_6446_MOESM1_ESM.docx]

**Supplementary Table 1.** Reliability parameters for interrater and intrarater agreement of water signal fraction measurement on T1-weighted Dixon images

|  | Interrater agreement (n = 25) | | | | |  | Intrarater agreement (n = 25) | | | | |
| --- | --- | --- | --- | --- | --- | --- | --- | --- | --- | --- | --- |
|  | ICC | | Paired-differences | | CV (%) |  | ICC | | Paired-differences | | CV (%) |
| **Radius** |  |  |  |  |  |  |  |  |  |  |  |
| ROI 1 | 0.79 | (0.85-0.90) | 0.08 | ± 6.4 | 27.7 |  | 0.97 | (0.93-0.99) | 0.7 | ± 2.6 | 10.9 |
| ROI 2 | 0.99 | (0.98-1.0) | 0.01 | ± 0.7 | 5.2 |  | 0.99 | (0.98-1.0) | 0.08 | ± 0.7 | 4.9 |
| ROI 3 | 0.96 | 0.90-0.98) | 0.1 | ± 1.0 | 9.4 |  | 0.98 | (0.96-0.99) | 0.04 | ± 0.7 | 6.1 |
| ROI 4 | 0.99 | (0.99-1.0) | 0.01 | ± 0.3 | 2.6 |  | 0.98 | (0.95-0.99) | 0.07 | ± 0.5 | 4.7 |
| ROI 5 | 0.99 | (0.98-1.0) | 0.07 | ± 0.3 | 3.1 |  | 0.98 | (0.95-0.99) | 0.1 | ± 0.5 | 4.6 |
| ROI 6 | 0.96 | (0.85-0.97) | 1.3 | ± 2.5 | 10.9 |  | 0.94 | (0.87-0.98) | 0.9 | ± 2.4 | 10.2 |
| ROI 7 | 0.98 | (0.95-0.99) | 0.06 | ± 1.4 | 9.7 |  | 0.98 | (0.94-0.99) | 0.3 | ± 1.5 | 10.3 |
| ROI 8 | 0.94 | (0.86-0.97) | 0.1 | ± 4.9 | 20.3 |  | 0.99 | (0.97-0.99) | 0.9 | ± 2.4 | 9.5 |
| ROI 9 | 0.99 | (0.98-1.0) | 0.09 | ± 1.0 | 6.9 |  | 0.99 | (0.98-1.0) | 0.4 | ± 0.7 | 5.1 |
|  |  |  |  |  |  |  |  |  |  |  |  |
| **Ulna** |  |  |  |  |  |  |  |  |  |  |  |
| ROI 10 | 0.86 | (0.67-0.94) | 1.5 | ± 3.2 | 13.5 |  | 0.91 | (0.78-0.96) | 1.1 | ± 2.4 | 9.8 |
| ROI 11 | 0.97 | (0.92-0.99) | 0.5 | ± 0.9 | 5.8 |  | 1.0 | (0.99-1.0) | 0.1 | ± 0.3 | 2.1 |
| ROI 12 | 0.96 | (0.89-0.98) | 0.4 | ± 0.9 | 7.5 |  | 0.99 | (0.99-1.0) | 0.09 | ± 0.3 | 2.8 |
| ROI 13 | 0.96 | (0.90-0.98)^*^ | 0.3 | ± 0.8 | 7.7 |  | 0.99 | (0.98-1.0)^*^ | 0.1 | ± 0.3 | 3.0 |

Data are presented as mean with (95 % confidence interval) or as mean ± standard deviation.

ROI, region of interest; ICC, intraclass correlation coefficient.
^*^ One case was excluded because of partial overlap of one or more ROIs with metaphyseal cortex.

**Supplementary Table 2.** Reliability parameters for interrater and intrarater agreement of water signal fraction measurement on T2-weighted Dixon images

|  | Interrater agreement (n = 25) | | | | |  | Intrarater agreement (n = 25) | | | | |
| --- | --- | --- | --- | --- | --- | --- | --- | --- | --- | --- | --- |
|  | ICC | | Paired-differences | | CV (%) |  | ICC | | Paired-differences | | CV (%) |
| ROI 1 | 0.88 | (0.74-0.94) | 0.2 | ± 6.3 | 19.7 |  | 0.88 | (0.75-0.95) | 0.2 | ± 6.2 | 18.7 |
| ROI 2 | 1.0 | (0.99-1.0) | 0.1 | ± 0.8 | 4.4 |  | 1.0 | (1.0-1.0) | 0.2 | ± 0.7 | 3.8 |
| ROI 3 | 0.99 | (0.98-1.0) | 0.2 | ± 1.0 | 6.7 |  | 1.0 | (0.99-1.0) | 0.05 | ± 0.8 | 5.0 |
| ROI 4 | 1.0 | (1.0-1.0) | 0.1 | ± 0.2 | 1.9 |  | 1.0 | (0.99-1.0) | 0.2 | ± 1.1 | 8.3 |
| ROI 5 | 0.99 | (0.98-1.0) | 0.1 | ± 0.5 | 3.9 |  | 0.98 | (0.96-0.99) | 0.3 | ± 1.2 | 9.3 |
| ROI 6 | 0.96 | (0.92-0.98) | 0.5 | ± 2.9 | 8.8 |  | 0.99 | (0.97-0.99) | 0.4 | ± 2.2 | 6.3 |
| ROI 7 | 0.99 | (0.98-1.0)^*^ | 0.1 | ± 1.3 | 6.4 |  | 1.0 | (0.99-1.0)^*^ | 0.4 | ± 1.6 | 7.1 |
| ROI 8 | 0.98 | (0.95-0.99) | 0.4 | ± 3.4 | 10.6 |  | 0.96 | (0.91-0.98) | 1.0 | ± 4.9 | 14.3 |
| ROI 9 | 0.99 | (0.98-1.0)^*^ | 0.4 | ± 1.5 | 7.6 |  | 1.0 | (0.99-1.0)^*^ | 0.1 | ± 0.9 | 4.4 |
|  |  |  |  |  |  |  |  |  |  |  |  |
| **Ulna** |  |  |  |  |  |  |  |  |  |  |  |
| ROI 10 | 0.92 | (0.82-0.97) | 1.1 | ± 3.5 | 10.8 |  | 0.94 | (0.85-0.98) | 2.0 | ± 4.0 | 11.7 |
| ROI 11 | 0.98 | (0.96-0.99) | 0.5 | ± 1.4 | 6.8 |  | 0.97 | (0.94-0.99) | 0.3 | ± 1.8 | 9.1 |
| ROI 12 | 0.97 | (0.93-0.99) | 0.5 | ± 1.4 | 8.7 |  | 0.93 | (0.84-0.97) | 0.02 | ± 1.9 | 12.8 |
| ROI 13 | 0.97 | (0.93-0.99)^**^ | 0.3 | ± 1.3 | 9.1 |  | 0.93 | (0.84-0.97)^**^ | 0.5 | ± 1.7 | 13.3 |

Data are presented as mean ± standard deviation or as mean with (95 % confidence interval).

ROI, region of interest; ICC, intraclass correlation coefficient; CV coefficient of variation.
^*^ 1 case was excluded because of partial overlap of one or more ROIs with metaphyseal cortex.

^**^ 2 cases were excluded because of partial overlap of one or more ROIs with metaphyseal cortex.

**Supplementary Table 3.** Intraclass correlation coefficients for agreement of water signal fraction between the three middle slices of the radius per sequence, measured by one observer

|  | T1-weighted Dixon (n = 66) | |  | T2-weighted Dixon (n = 66) | |
| --- | --- | --- | --- | --- | --- |
| ROI 1 | 0.81 | (0.73-0.87) |  | 0.80 | (0.72-0.87) |
| ROI 2 | 0.92 | (0.88-0.95) |  | 0.93 | (0.90-0.95) |
| ROI 3 | 0.87 | (0.81-0.91) |  | 0.90 | (0.85-0.93) |
| ROI 4 | 0.71 | (0.60-0.80)^*^ |  | 0.76 | (0.66-0.83) |
| ROI 5 | 0.55 | (0.41-0.68) ^†^ |  | 0.70 | (0.58-0.79) ^†^ |
| ROI 6 | 0.84 | (0.77-0.89) |  | 0.89 | (0.84-0.93) |
| ROI 7 | 0.94 | (0.92-0.96)^*^ |  | 0.96 | (0.94-0.97)^**^ |
| ROI 8 | 0.91 | (0.88-0.94) |  | 0.94 | (0.90-0.96) |
| ROI 9 | 0.94 | (0.91-0.96)^*^ |  | 0.95 | (0.92-0.97)^**^ |

Data are presented as mean with (95 % confidence interval).

ROI, region of interest.

^*^ One case was excluded because of partial overlap with metaphyseal cortex.

^†^ Four cases were excluded because of partial overlap with metaphyseal cortex

^**^ Three cases were excluded because of partial overlap with metaphyseal cortex.

**Supplementary Table 4.** Intraclass correlation coefficients for absolute agreement between ROIs 1 and 2 and mean of three ROIs measured at the same distance to the physis

|  | T1-weighted Dixon | |  | T2-weighted Dixon | |
| --- | --- | --- | --- | --- | --- |
| ROI 1 and mean of ROIs 1, 6 and 8 | 0.97 | (0.95-0.98) |  | 0.96 | (0.94-0.98) |
| ROI 2 and mean of ROIs 2, 7 and 9 | 0.95 | (0.89-0.98)^*^ |  | 0.97 | (0.93-0.98)^*^ |

Data are presented as mean with (95 % confidence interval).

ROI, region of interest; ICC, intraclass correlation coefficient; CI, confidence interval.

^*^ One case was excluded because of partial overlap with metaphyseal cortex.

**
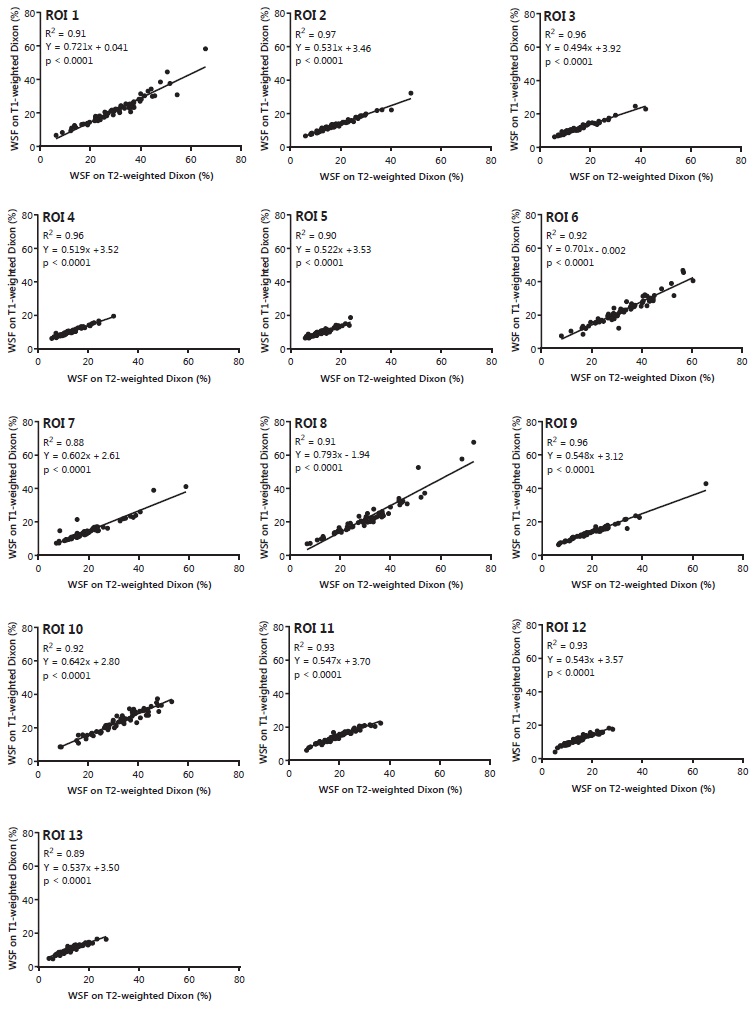
**

**Supplementary figure 1.** Results of linear regression between water signal fraction measured on T1-weighted and T2-weighted Dixon images, calculated per region of interest. WSF, water signal fraction (%); ROI, region of interest.
